# C.I. Acid Black 1 transfer from dilute solution to perlite framework in organic waste management

**DOI:** 10.1007/s10653-024-02013-3

**Published:** 2024-06-07

**Authors:** Maria Roulia, Alexandros A. Vassiliadis

**Affiliations:** 1 Inorganic Chemistry Laboratory, Department of Chemistry, National and Kapodistrian University of Athens, Panepistimiopolis, 157 71 Athens, Greece; 2 Dyeing, Finishing, Dyestuffs and Advanced Polymers Laboratory, DIDPE, University of West Attica, 250 Thivon St., 122 41 Athens, Greece

**Keywords:** Dye-contaminated land, C.I. Acid Black 1, Perlite, Adsorption, Isotherms, Soil chemistry, Hydrophobic–hydrophilic sites

## Abstract

Dyes, considered as toxic and persistent pollutants, must be removed from organic wastes prior to their composting and application in sustainable agriculture. Azo dyes, capable of altering the physicochemical properties of soil, are difficult to expel by conventional wastewater treatments. C.I. Acid Black 1 (AB 1), a sulfonated azo dye, inhibits nitrification and ammonification in the soil, lessens the nitrogen use efficacy in crop production and passes substantially unaltered through an activated sludge process. The retention of C.I. Acid Black 1 by raw and expanded perlite was investigated in order to examine the potential effectiveness of this aluminosilicate material toward organic waste cleanup. Dye adsorption proved spontaneous and endothermic in nature, increasing with temperature for both perlites. Expanded perlite having a more open structure exhibited a better performance compared to the raw material. Several of the most widely recognized two-parameter theoretical models, i.e., Langmuir, Freundlich, Temkin, Brunauer–Emmett–Teller (BET), Harkins–Jura, Halsey, Henderson, and Smith, were applied to reveal physicochemical features characterizing the adsorption. The Langmuir, Freundlich, Temkin, BET, Henderson, and Smith equations best fitted experimental data indicating that the adsorption of anionic dye on perlites is controlled by their surface, i.e., non-uniformity in structure and charge. This heterogeneity of surface is considered responsible for promoting specific dye adsorption areas creating dye “islands” with local dye supersaturations.

## Introduction

Organic wastes can be biodegraded under controlled conditions (e.g., acidity, temperature, moisture) (Kazamias et al., 2017) via composting, a natural biological decomposition method of recycling, in which decomposer microorganisms break down organic compounds into small molecules; organic waste may contain disposed textile (Biyada et al., 2021) and leather (Miranda et al., 2019) dyed products, as well as azo dyes used in soaps, cosmetics and pharmaceuticals.

During composting, water-soluble synthetic dyes migrate from the organic solid to an aqueous phase. As these dyes are generally toxic, persistent pollutants and the resulting dye-loaded solution is neither bio- nor photodegradable, the compost is not suitable for sustainable agricultural practices if applied directly to the soil. Hence, organic azo dyes must be retained on a water-insoluble substrate, e.g., an adsorbing material, by other than merely mechanical means (Patermarakis & Vassiliadis, 2023; Roulia & Vassiliadis, 2005, 2008) to obtain a quality compost.

Likewise, colored wastewater thus treated is commonly used in semi-arid regions as irrigation water for agriculture in order to overcome water shortage. Substances present in textile dyeings and finishes, as well as dyes from various classes, are lost in exhaust dyebaths and wash liquors. Residues of the dyeing processes, e.g., azo dyes capable of being reduced to form hazardous arylamines, may create water pollution problems after their discharge into the environment. The main consideration regarding the environmental impact of residual dyes is concerned with toxicity to organisms and soils. Agricultural crops absorb organic dyes from the soil, the dyes are transported from soils to plants and can inhibit root growth, damage root tips, lower the uptake of nutrients and water, reduce germination and photosynthetic pigments (Zhou, 2001). As only low levels of these dyes can be removed by the activated sludge process (Kandelbauer et al., 2007), when organic dyes (commonly, synthetic aromatic compounds) are to be removed from textile wastewater, adsorption is an inexpensive useful technique (Angelova et al., 2017; Bensalah, 2024; George et al., 2024; Khamis et al., 2024) and perlites (Mathew et al., 2018; Painer et al., 2022) are abundant, economically attractive adsorbents (Khoshraftar et al., 2023; Roulia & Vassiliadis, 2005, 2008). Decolorized dyebath wastewaters (Roulia & Vassiliadis, 2021) with a nitrogen content reduced to acceptable levels meet the quality standards for surface and ground water and could effectively be applied to crops.

Azo dyes contain at least one diazene group attached to, usually, two aromatic rings that are trans to each other (as the nitrogen atoms are sp^2^-hybridized, the bond angles are about 120°). Water solubility can be conferred upon an azo dye by sulfonic groups, commonly as the sodium salt of the free acid. These anionic dyes, bearing a negative charge, are typically applied to either natural (wool, silk, leather) or synthetic (e.g., Nylon^®^) polymers from an acid-containing aqueous bath, are also known as acid dyes and form ionic bonds within the cationic polymer matrix as shown in Eq. (1)

1 $$ {{Dye}} - {\text{SO}}_{3}^{( - )} {\text{Na}}^{( + )} + {{Polymer}} - {\text{NH}}_{3}^{ + } \to {{Dye}} - {\text{SO}}_{3}^{( - )( + )} {\text{H}}_{3} {\text{N}} - {{Polymer}} + {\text{ Na}}^{ + } $$

where *Polymer* is a protein (keratin, fibroin) or a linear polyamide. On completion of dyeing, the dyestuff that resides within the solution phase, released into water streams used for irrigation, negatively affects the crops and agricultural plants on a reasonably large scale as already mentioned. If allowed to flow into the fields, textile wastes are deleterious to crops; azo dyes destroy plant beneficial bacteria habitats in soil (Krishnamoorthy et al., 2021) and suppress the growth of plant rhizobacteria (Imran et al., 2015). Actually, it is a highly critical issue to remove dyes from the aqueous effluent (Georgiou et al., 2017).

C.I. Acid Black 1 (C.I. 20470) is a water-soluble dyestuff (1–5 g dye in 100 g water, absorbance maximum (He & Hu, 2011) at λ_max_ = 618 nm), bluish-black –in daylight and water– or deep blue –in ethanol–, particularly suitable for dyeing protein fibers, leather (Sivakumar et al., 2005), widely used in cellulosics (paper, wood stains) (Galán et al., 2013; Gordon & Gregory, 1987), metals (anodized aluminum), organic materials (casein buttons, urea–melamine moulding powders), colorants (leather dyestuffs, writing inks, hair non-oxidative dyes), pharmaceuticals (drugs, soaps, personal care products) and as a biological protein-staining reagent.

In this work, the removal of C.I. Acid Black 1 (AB 1) from aqueous solutions by adsorption onto raw and expanded perlites is studied and the applicability of eight adsorption isotherms is investigated in an attempt to determine the architecture of organic dye–aluminosilicate matrix nanocomposites and the adsorption effectiveness of framework edge-sites on perlite. Toxicity of this dye to soil microorganisms and to agriculturally significant processes of nutrient cycling can, thus, be avoided.

## Materials and methods

### Materials

Raw perlite, a chemically inert glassy volcanic aluminosilicate rock, has a density of 1.1 kg L^−1^ and contains 2–6 wt% trapped, encapsulated water. The perlite ore softens and expands upon rapid heating at 970–1470 K. Expanded perlite is frothy, hydrophilic and environmentally safe, is characterized by sterility, low density (0.03–0.15 kg L^−1^) and a neutral pH, and is composed of tiny irregular shreds, clusters of trapped bubbles, closed air cells, surface broken openings and minute cavities that offer the material an extensive surface area and high porosity, thus, increasing its absorption capacity (Roulia & Vassiliadis, 2005; Roulia et al., 2014). Raw and expanded perlite (Silver and Baryte Ores S.A., Greece) originated from the Tsigrado region, Milos island, Greece. All perlite samples were first crushed to pass a 1-mm sieve. The chemical composition of raw perlite has been reported elsewhere (Roulia & Vassiliadis, 2005).

**Fig. 1:**
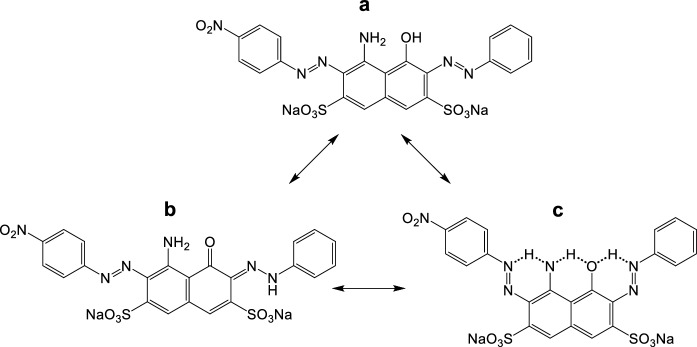
Azo (**a**) and hydrazone (**b**) structural formulae of C.I. Acid Black 1 (M_r_ = 616.49 Da); hydrogen bonds (**c**) in the dye molecule

The diazo acid dye disodium (6Z)-4-amino-3-(4-nitrophenyl)diazenyl-5-oxo-6-(phenylhydrazinylidene)naphthalene-2,7-disulfonate (C.I. Acid Black 1), presented in Fig. 1, exists in two tautomeric forms, the azo (Fig. 1a) and the strongest hydrazone dye (Fig. 1b). The two sulfonate groups ensure that the dye is overall anionic, with isoelectric point 4.52, a density of 1.05 g mL^−1^ at 293 K, melting point > 620 K and estimated molecular size 21 × 12 × 6 Å (He & Hu, 2011) or 22.9 × 11.2 × 5.44 Å (Zhao et al., 2013).

Intramolecular hydrogen-bonding exists (Fig. 1c) that enhances the stability of AB 1 and confers good lightfastness properties as it leads to a reduction in electron density at the chromophore and improves photostability, decreasing the sensitivity of the dye toward photochemically-induced oxidation. In excited-state intramolecular proton transfer [Smith et al., 1991] the nitrogen atom acts as the proton donor. The intramolecular hydrogen bonds reduce the acidity of the hydroxyl group and offer a significantly improved resistance to alkaline conditions. Photocatalytic decoloration of C.I. Acid Black 1 (i.e., Amido Black 10B) has been investigated over lithium niobate and doped lithium niobate powders (Stock & Dunn, 2012), using a heterostructured Bi_2_O_3_–ZnO semiconductor photocatalyst (Balachandran & Swaminathan, 2012), in the presence of composed Tl_4_CdI_6_ nanoparticles (Ghanbari & Salavati-Niasari, 2018), and by adsorption on a SiO_2_@TiO_2_/CuBiS_2_/Ag composite catalyst (Abdullah & Kuo, 2015). Additionally, C.I. Acid Black 1 has been treated by ozone oxidation in the presence of chlorides that interfere considerably with dye degradation (Paprocki et al., 2010), has been degraded and used as a nitrogen source by marine cyanobacteria (Priya et al., 2011), and has been removed via biosorption by algae biomass (Kousha et al., 2012).

A strain isolated from soil, i.e., *Bacillus cereus*, has been used (Wuhrmann et al., 1980) for the microbial reduction of C.I. Acid Black 1 (i.e., Naphtholblauschwarz E); permeation of AB 1 through the cell wall is the decoloration determining step but the sulfonic acid substitution is an effective permeation inhibitor as the reactive sulfonate groups in the molecule (Fig. 1) make the dye xenobiotic. Dyeing discharge containing AB 1 fails to meet NH_3_ and BOD_5_ limits at low temperatures and increased dye concentrations; complete nitrification failure has been observed (Martin et al., 2005) in a wastewater treatment plant. In principle, arginine ammonification, nitrification, ammonium and nitrite oxidizing bacteria in azo-dye polluted soil depend primarily on the dye content.

Adsorption-decolorized textile sludge that contains many organic compounds, improves soil properties, acts as soil conditioner and is rich in plant nutrients (i.e., nitrogen, phosphorus, potassium) may be used in agriculture as an alternative to chemical fertilizers. Owing to its high sulfonic acid content, C.I. Acid Black 1 has been found to pass through activated sludge without any biodegradation or adsorption on it (Martin et al., 2005; Shaul et al., 1991; Wuhrmann et al., 1980). The adsorption of dye on a granular (Hadi et al., 2010) and a lignocellulosic waste biomass (Nethaji & Sivasamy, 2011) activated carbons has been measured, ranging between 400–450 and 2.4–4.0 mg dye/g adsorbent, respectively. Experimental adsorption capacities of a compost (Kyzioł-Komosińska et al., 2011) 0.018–12.74 mg g^−1^, of peat (Sepúlveda et al., 2004) 25 mg g^−1^, of fly ash (Sun et al., 2010) 35 mg g^−1^, and of bentonite (Pająk et al., 2019) 31.29 mg g^−1^, have been determined. Values for the dye retention by mesoporous carbons have been reported as 270 (Galán et al., 2013) and 271.39–497.93 mg g^−1^ (Peng et al., 2014). The maximum adsorption capacities of a cross-linked chitosan/bentonite composite (Liu et al., 2015), chitosan aerogels doped with graphene oxide (Wang et al., 2015) and polyamidoamine-grafted chitosan (Banisheykholeslami et al., 2021) have been 323.6–350.9, 573.47 and 543.2 mg g^−1^, respectively. The adsorption capacity of a cationic metal organic framework (Zhao et al., 2019) has been estimated as being 419 mg g^−1^.

### Experimental

**Fig. 2:**
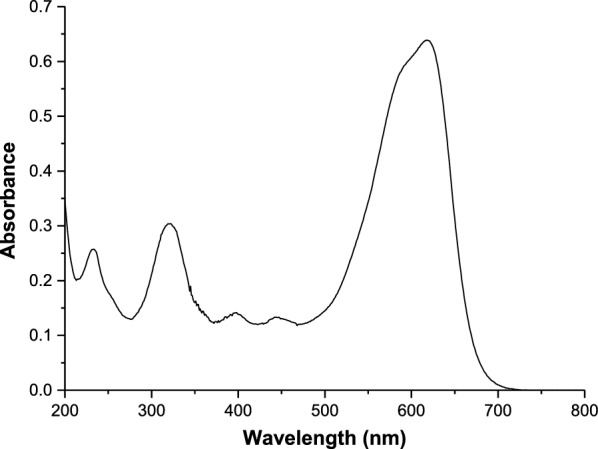
UV–vis spectrum of C.I. Acid Black 1 (AB 1)

C.I. Acid Black 1 (Chromatourgia Tripoleos S.A., Greece) absorbs visible light with *λ*_max_ at 618 nm (Fig. 2). A linear calibration curve was established by plotting absorbance at *λ*_max_ against dyestuff concentration; all dye contents were derived from this curve. Absorbances in the visible range were measured on a Varian Cary 3E UV–vis spectrophotometer.

The dye–perlite interactions were studied in aqueous solutions of five initial dye concentrations, *C*_*o*_ (i.e., 0.02, 0.04, 0.06, 0.08 and 0.10 g L^−1^). 50 mL of each dyestuff solution and 0.5 g of either raw or expanded perlite were placed in a sealed stainless steel vessel and dye–aluminosilicate mixtures, with dye loadings between 0.02 and 0.10 g L^−1^ (0.0324 and 0.162 mmol L^−1^, respectively), were treated at a circulation rate of 30 min^−1^ in a programmable laboratory dyeing machine (Atlas Linitest plus) for 3 h at constant pH and temperature. The pH range of the dispersions was 6.5–8.0; dye retention measurements were made at 323, 353, and 383 K. Samples of dyed perlite were separated from the residual dye solution by centrifugation in a Martin Christ Osterode/Harz centrifuge (5000 min^−1^, 30 min).

The point of zero charge (pH_PZC_) was measured according to the pH drift method (Jia et al., 2002). Specifically, the pH value of a 50 mL 0.01 M NaCl solution was adjusted between 2 and 12 using 0.1 M solutions of either hydrochloric acid or sodium hydroxide. To avoid pH changes due to dissolved CO_2_, N_2(g)_ was bubbled through the NaCl solution. Then 0.5 g perlite (raw or expanded) were added to the solution, the mixture was stirred for 24 h until pH stabilized and the final pH value was recorded. The pH_PZC_ was determined as the point where the initial and final pH values equal. A Jenway 3310 pHmeter was employed to measure the pH of the dispersions.

Scanning electron microscopy (SEM) has proved especially useful for studying the surfaces of fine-grained materials and most suitable for the examination of perlite configuration (particle size and shape). Scanning electron micrographs of untreated raw perlite were taken with an environmental scanning electron microscope (XL/30 ESEM Philips). Gold-coated samples of dyed raw perlite and heat-modified expanded perlite were examined in a Jeol JSM-5600 scanning electron microscope.

## Results and discussion

### Equilibrium adsorption

**Fig. 3:**
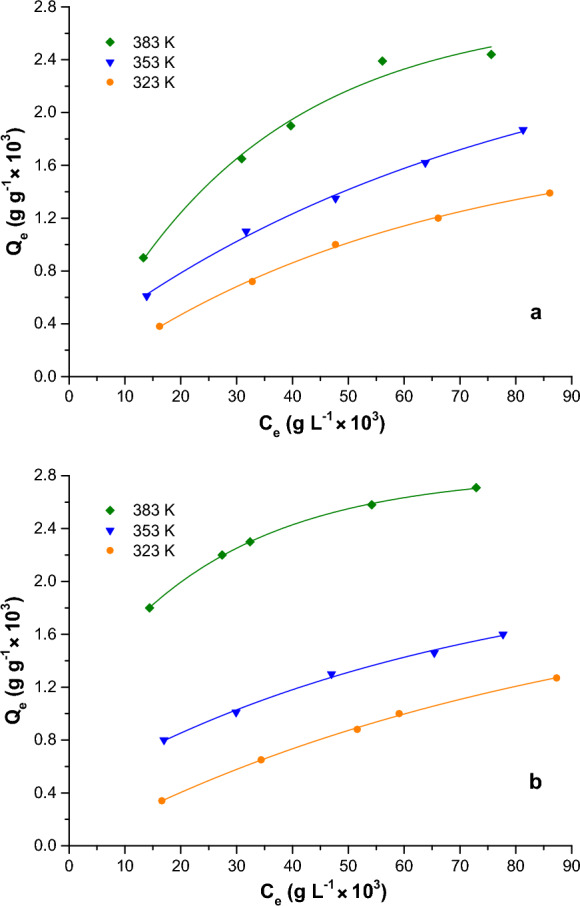
Adsorption isotherms of C.I. Acid Black 1 onto raw (**a**) and expanded (**b**) perlite

The equilibrium dye contents, *Q*_*e*_, are plotted against equilibrium dyestuff concentrations in solution, *C*_*e*_, at 323, 353, and 383 K for both raw and expanded perlite in Fig. 3a, b, respectively. All are “Langmuir” isotherms, suggesting monolayers of surface-adsorbed dye molecules. Adsorption capacity of both perlites increases with temperature; this is an activated in the reaction-kinetic sense process as a high energy barrier has to be overcome by the adsorbate molecules before adsorption occurs.

Low rates of adsorption are probably due to the like charges of adsorbent and adsorbate, to the diversity of adsorption sites, resulting from the non-uniform structure of perlite, to the action of water molecules that reduce the energy of the surface by competition with the solute, as well as to the diffusion of dye molecules through narrow constrictions into cavities beyond. On the other hand, increased dye mobility enables the dye anions to insert as temperature increases, even though the dye-ion localization onto perlite is expected to decrease (Giles et al., 1960; Gregg & Sing, 1982; Roulia & Vassiliadis, 2008).

### Ιsotherm equations

Eight two-parameter adsorption models (Table 1) were fit to the experimental data. The Langmuir, Freundlich, Temkin, BET, Harkins–Jura, Halsey, Henderson, and Smith isotherm equations can all reveal distinct adsorption-related physicochemical characteristics of perlites.

**Table 1:** Linearity coefficients of the theoretical models

Isotherm	Temperature (K)	R^2^	
		Raw perlite	Expanded perlite
Langmuir $\frac{{C_{e} }}{{Q_{e} }} = \frac{1}{{KQ_{m} }} + \frac{1}{{Q_{m} }}C_{e}$	323	0.964	0.990
	353	0.988	0.990
	383	0.974	1.000
Freundlich $\log Q_{e} = \log K_{F} + \frac{1}{n}\log C_{e}$	323	0.988	0.995
	353	0.996	0.997
	383	0.970	0.985
Temkin $Q_{e} = \frac{RT}{b}\ln A + \frac{RT}{b}\ln C_{e}$	323	0.992	0.981
	353	0.984	0.987
	383	0.981	0.996
BET $\frac{{C_{e} }}{{Q_{e} \left( {1 - C_{e} } \right)}} = \frac{1}{{X_{m} K_{B} }} + \frac{{K_{B} - 1}}{{X_{m} K_{B} }}C_{e}$	323	0.968	0.990
	353	0.993	0.994
	383	0.973	1.000
Harkins–Jura $\frac{1}{{Q_{e}^{2} }} = \frac{B}{A} - \frac{1}{A}\log C_{e}$	323	0.851	0.869
	353	0.898	0.969
	383	0.873	0.940
Halsey $\ln Q_{e} = \frac{1}{n}\ln k - \frac{1}{n}\ln (\ln \frac{1}{{C_{e} }})$	323	0.965	0.977
	353	0.982	0.992
	383	0.938	0.961
Henderson $\ln \left[ { - \ln (1 - C_{e} )} \right] = \ln k + n\ln Q_{e}$	323	0.987	0.994
	353	0.996	0.996
	383	0.969	0.983
Smith $Q_{e} = W_{b} - W\ln (1 - C_{e} )$	323	0.964	0.978
	353	0.978	0.981
	383	0.884	0.907

The Langmuir theory (Langmuir, 1918) provides a good equation to mathematically describe the adsorption and its fit suggests the formation of a dye monolayer. For a uniform surface all adsorption sites are equivalent, according to the Langmuir concept, with no interactions between the adsorbed species. However, there are adsorbing surfaces of a high non-uniformity (Halsey, 1948) and original substrates in which only a minor surface portion may be available for adsorption, both obeying the Langmuir isotherm.

The calculated values of the Langmuir adsorption constant, *K*, increase with temperature revealing that C.I. Acid Black 1 adsorption onto perlites is a temperature-dependent process. On the other hand, the monolayer adsorption capacity, *Q*_*m*_, remains practically constant (0.0035 g g^−1^) for both adsorbents and accords well with the observed adsorption capacity of perlites. Given the significant difference (Roulia & Vassiliadis, 2008) in the CEC value (25 mEq/100 g raw and 35 mEq/100 g expanded perlite), this observation may be associated with the absence of Coulombic interactions between the adsorbate and the substrate.

The solution adsorption isotherms of both perlites perfectly fit the Freundlich equation (Freundlich, 1907), a basic principle of which is the heterogeneous distribution of the adsorption sites; thus, the adsorbing surface becomes covered with unevenly arranged molecules of the heterogeneously adsorbed dye and promotes the development of local dye supersaturations. It would seem logical that adsorption takes place only on a few highly active sites of perlites.

The surface charge density values, derived from the CEC and the total surface area (TSA), were 0.192 and 0.0745 mEq m^−2^ (Roulia & Vassiliadis, 2008) for raw and expanded perlite, respectively; their total surface (1.30 and 4.70 m^2^ g^−1^, respectively) coincides with the BET surface area (Roulia & Vassiliadis, 2005). Although raw perlite possesses a considerably greater surface charge density compared with expanded, its adsorption capacity shown in Fig. 3 is just relatively lower. As described above, the dye –when adsorbed onto the surface of perlites– forms high-concentration patches on the active adsorption locations; surface charge density may represent the “mass-penetration” degree (Roulia & Vassiliadis, 2008) achieved by AB 1.

The Temkin isotherm (Cerofolini & Rudziński, 1997; Temkin & Pyzhev, 1940) suggests a heat of adsorption to binding interactions relationship and involves a uniform distribution (Qurrat-ul-Ain et al., 2019) of adsorbing–adsorbate binding energies (occurring due to the even non-uniformity of the surface). This should be contrasted to Langmuir model that assumes a single binding energy. The logarithmic Temkin equation applies to medium surface coverages. By the quasi-logarithmic equation, valid for a full range of coverages, the fraction of the surface covered by the adsorbate, *θ*, is given as a function of *α*_0_, *α*_1_ (the adsorption coefficients for the strongest and weakest sites of adsorption, respectively) and* f* (a parameter of the linear energy distribution function of adsorption sites) representing the surface non-uniformity,

2 $$f = \ln \left( {{{a_{0} } \mathord{\left/ {\vphantom {{a_{0} } {a_{1} }}} \right. \kern-0pt} {a_{1} }}} \right) = {{\left( {Q_{\max } {-} \, Q_{\min } } \right)} \mathord{\left/ {\vphantom {{\left( {Q_{\max } {-} \, Q_{\min } } \right)} {{\text{RT}}}}} \right. \kern-0pt} {{RT}}}{\text{,}}$$

where *Q* is the adsorbate binding energy. On a strongly heterogeneous surface *a*_1_ differs significantly from *a*_0_, *f* is substantially greater than unity and the probabilities of being occupied the most strongly and the most weakly adsorbing sites are ~ 1 and ~ 0, respectively. In a multicomponent adsorption system of *n* components, the binding energies of components with the surface would change in different ways on adsorption sites of different types (Murzin, 2019; Temkin, 1979; Tovbin, 2019). Heterogeneity of binding seems to be the key parameter to justify the fit of experimental data to this isotherm equation for perlites.

The BET equation (Brunauer et al., 1938) yields an excellent fit to the AB 1 adsorption data for both perlites which was unexpected as this model, firstly, is based on the formation of adsorbate multilayers; and secondly, similarly to the Langmuir isotherm, is applicable to homogeneous adsorption. The observation that perlites follow the BET equation in spite of their highly non-uniform adsorption surface can be explained by the fact that at low adsorbate concentrations the BET equation reduces to the Langmuir isotherm which quite well fitted experimental data. The BET isotherm theory indicates that condensation begins when the number of molecules adsorbed equals the value required for a complete monolayer. As the first stages of condensation are connected with preferential adsorption on strongly attracting sites, the applicability of BET model to the perlites may result from the patchwise topography of their surface that favors the creation of supersaturated-dye “islands”.

In the Harkins–Jura isotherm equation (Harkins & Jura, 1944) it is assumed that the adsorbed molecules form a condensed film. Compared to the BET model, this isotherm has a linear region more extensive, corresponding to higher adsorbate concentrations. This is the reason why relatively low values of correlation coefficient were estimated and the quality of fit falls off for both perlites (Table 1). These can also explain in part the obtained fit to experimental data for expanded perlite (which exhibits a relatively higher dye adsorption with increasing temperature).

Both the Harkins–Jura and Halsey (Halsey, 1948) equations incorporate the assumption that the distribution of active sites is non-uniform, and this is the case when adsorption on perlites is investigated. According to the Halsey theory the number of neighboring molecules being adsorbed simultaneously (Halsey, 1948) may become large enough to cause cooperative condensation. A very good fitting of the experimental data was achieved as outlined in Table 1, and this can be ascribed to that the adsorption of C.I. Acid Black 1 is controlled by the heterogeneous surface of perlites and involves dye interactions in the cooperative sense. Thus, the observed behavior appears to be in accord with the Halsey model.

The Henderson empirical isotherm reflects the nature of moisture adsorption (Henderson, 1952) onto hygroscopic materials –including amorphous anisotropic aluminosilicates– by considering a mainly physical adsorbent–adsorbate interaction and a multilayering arrangement of the water adsorbed. The quantity of adsorbate is a function of the adsorbing surface and, provided that the assumptions made in deriving the Henderson equation are satisfied, the surface heterogeneity could influence the mechanism of adsorption. Thus, the fact that both dye-treated perlites satisfy the Henderson equation is attributable to building-up of adsorbed solute molecules on perlite heterogeneous surface.

The Smith treatment (Smith, 1947) subdivides the weight fraction of adsorbed substance into a bound and a normally condensed fraction. There is a remarkable similarity between the mathematical bases of both Smith and BET isotherms that differ materially in one respect; in the BET theory, the contents of the second and all higher layers are related to each other and to the content of the first layer (Smith, 1947). Although these models start from a different concept, there appears to be a concordance between the characteristic values of the BET equation and that of Smith, demonstrating why both perlites obey the Smith isotherm (Table 1). A second feature notable in Table 1, however, is that in this case a better fit to experimental data is provided with decreasing temperature.

Most of the isotherm equations proved equally applicable to the dye–perlite pair as the adsorption picture is consistent with multiple adsorbing principles. With one exception (Harkins–Jura), and the possible exception of the Halsey and Smith models at the highest temperature (383 K), all isotherms employed gave a close fit to the measured data with the most successful being the Temkin and Henderson models. It is evident from Table 1 that the Langmuir, Freundlich and BET equations were quite successfully applied to expanded perlite. Table 1 also shows that, in almost all cases, a better fit is obtained for expanded perlite. This may be due to the nature of expanded perlite, which is more homogeneous than is the raw aluminosilicate (Roulia et al., 2006, 2014) as the condensation of framework and the rejection of non-adsorbing admixtures that both occur during expansion increase the homogeneity of perlite. It seems that the surface of unexpanded material is excessively heterogeneous, contrary to what is assumed in the models used.

Deviations from straight line (Halsey and Smith isotherms) demonstrate the limitations of these models as the temperature is raised. In this case, the increased adsorbate mobility and the extensive delocalization between adjacent active sites both promote the adsorption at higher temperatures but induce a less satisfactory fit of the models (Gregg & Sing, 1982; Roulia & Vassiliadis, 2008) as elevated temperatures may, on the one hand, enhance adsorption capacity but, on the other, bring about changes in physicochemical parameters that have been taken into account in the isotherms.

### Adsorption thermodynamics

Important thermodynamic parameters, i.e., the enthalpy change, ${\Delta }H_{ads}^{o}$, and the entropy change, ${\Delta }S_{ads}^{o}$, were calculated (Table 2) by plotting ln*K* against 1/*T*, from the slope and the intercept, respectively, of the straight line using the van’t Hoff equation in the form (Lima et al., 2019)

3 $$\ln K = \frac{{\Delta S_{{{ads}}}^{o} }}{R} - \frac{{\Delta H_{{{ads}}}^{o} }}{{{RT}}}{\text{,}}$$

where *K* is the Langmuir equilibrium constant expressed in L mol^−1^. The Gibbs free energy of the adsorption, ${\Delta }G_{ads}^{o}$, is given by the relationship

4 $$\Delta G_{ads}^{o} = \Delta H_{ads}^{o} - T\Delta S_{ads}^{o}{\text{.}}$$

**Table 2:** Thermodynamic parameters (± 9%) for AB 1 adsorption on perlites

Adsorbent	Temperature (K)	$\Delta {H}_{ads}^{o}$ (kJ mol^−1^)	${\Delta }S_{ads}^{o}$ (J mol^−1^ K^−1^)	${\Delta }G_{ads}^{o}$ (kJ mol^−1^)
Raw perlite	323	18.9	186.6	−41.4
	353			−47.0
	383			−52.6
Expanded perlite	323	45.4	267.2	−40.9
	353			−48.9
	383			−56.9

The ${\Delta }H_{ads}^{o}$ data in Table 2 indicate endothermic events favored by the temperature raise. The values of ${\Delta }H_{ads}^{o}$ and ${\Delta }S_{ads}^{o}$ can rarely be attributed to a single process compiling all subprocesses involved in adsorption, e.g., conformational changes, retention mechanisms and equilibria including dehydration of both sorbent and dye (Sellergren & Shea, 1995). A greater enthalpy change for expanded perlite is in accordance with the increased adsorption observed at higher temperatures. The free-energy values (Table 2) underline that dye adsorption is spontaneous increasingly facilitated with temperature for both perlites.

### Acidity levels

The above mentioned decoloration or degradation of C.I. Acid Black 1 by a photocatalytic, oxidative or biological method (Abdullah & Kuo, 2015; Balachandran & Swaminathan, 2012; Ghanbari & Salavati-Niasari, 2018; Kousha et al., 2012; Paprocki et al., 2010; Priya et al., 2011; Stock & Dunn, 2012) is not likely to occur under actual running, bulk-scale operating conditions, where solid and liquid wastes have to be treated and discharged. Given that the approaches of either mixing municipal and industrial organic wastes (both biodegradable) prior to composting or source separating municipal waste (that is, separate collection of organics) are popularly adopted in many countries, a number of attractive possibilities are offered allowing the efficient adsorptive removal of hazardous pollutants (e.g., dyes) from biowaste. The acidity of wastes is of fundamental significance because it affects the surface reactivity of the adsorbent, as well as the form and charge of the dyestuff structure, i.e., the ionic properties in the dye molecule. Adjustment of pH, governing the transfer of dye from the aqueous to the solid phase, is also of critical importance. The determination of the isoelectric point of perlite framework edges through the pH_PZC_ (point of zero charge) measurement and, also, knowledge of the pH of dye solutions that were subjected to the adsorption experiments can both be used to tailor the effectiveness of the treatment and its practical application.

Using this particular adsorption process, C.I. Acid Black 1 removal from waste of anthropogenic origin, i.e., industry (e.g., textile dyeing processes, leather tanning) discharges and source-separated organic (SSO) biodegradable municipal wastes (e.g., skin-care products, hair colorants, synthetic detergents), can be achieved. Therefore, the existence of the particular azo dye (AB 1) in the final composted products, in irrigation waters and in agricultural soils could be entirely eliminated.

**Fig. 4:**
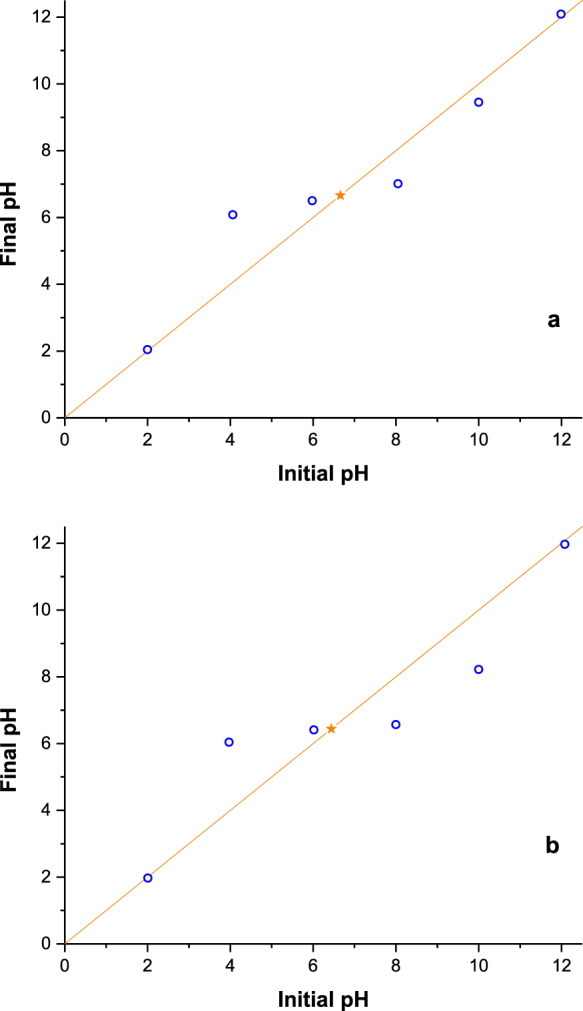
The pH_PZC_ of raw (**a**) and expanded (**b**) perlite

Figure 4a, b demonstrates the graphs of final pH versus initial pH obtained using the pH drift method for both materials. The results show that the pH_PZC_ for both raw and expanded perlite are quite similar, with the pH_PZC_ values being 6.66 and 6.44, respectively. In this context, the perlite’s surface will predominantly bear a positive charge at pH values lower than the pH_PZC_ while at pHs higher than the pH_PZC_ both perlites will appear mostly negatively charged; a 20% increase in AB 1 retention was observed at pHs 2 and 3, consistent with Fig. 4.

It should be noted, however, that perlite is in principle a negatively charged aluminosilicate (unbound terminal –SiO^–^ groups and isomorphous substitutions) and that the balancing cations are very labile, i.e., easily exchangeable. Thus, adsorption of AB 1 in patches onto perlite unravels, besides the structural diversity, the charge heterogeneity in perlite possibly accompanying the “broken-bond” or “edge” existing surfaces where the unsatisfied atoms may bring positive charges (Yariv & Cross, 1979).

### Surface morphology and texture

**Fig. 5:**
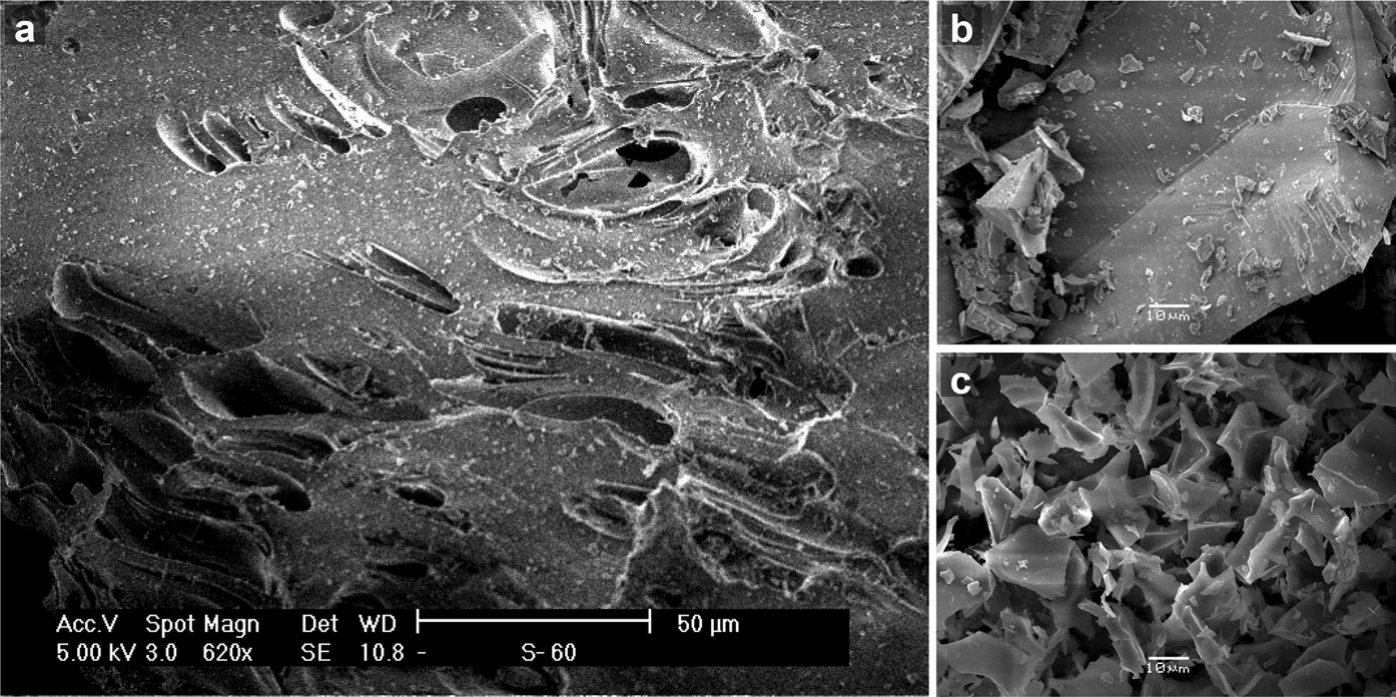
Scanning electron micrographs of host (**a**) and dye-treated (**b**) raw perlite, and of undyed expanded perlite (**c**); all three images are presented in such a manner that equal lengths shown in the micrographs correspond to nearly equal actual values, i.e., sizes of surface features, in the original samples

The micrograph of untreated raw perlite in Fig. 5a illustrates a typical smooth and even surface covered by particles 1 to 5 μm in size. The sample exhibits sparse scales; several tiny ovoid holes are also shown in the micrograph. Prismatic fragments are observed on the surface of dyed raw perlite at the left in the micrograph of Fig. 5b, which also depicts a number of relatively fine grains (1–8 μm). Raw perlites in Fig. 5a, b are similar in appearance and compacted into massive, featureless blocks without distinctive or characteristic parts. These two micrographs display insignificant differences with no evidence of penetration textures due to dye presence as shown by Fig. 5b.

Expanded perlite has a characteristic morphology of irregular small shreds randomly placed; as a result, an open structure is generated. A typical micrograph of expanded perlite (Fig. 5c) does show this configuration quite well. The polygonal fragmented pattern of expanded perlite, i.e., its overall particle arrangement that differs from the blocky texture of raw perlite, can be seen clearly in this micrograph. Expanded perlite exhibits no remarkable alterations after the dye adsorption; the micrograph of host expanded perlite in Fig. 5c presents essentially the same morphology as a micrograph of dye-treated sample.

### Dye impact on soils and agriculture

The textile processing industry is, in fact, the second major water consumer next to agriculture. The wet processes used in textile dyeing and finishing require a large amount of water. Land application of textile organic waste and crop irrigation waters polluted by dyehouse effluents provide the driving force for the movement of synthetic dyestuffs through agricultural soil. At a low flow rate, the dye–soil contact time is high and a bonding interaction between the dye molecules and soil particles is possible, resulting in an intensification of dye retention on soil materials (Jedidi et al., 2020; Smaranda et al., 2017); adsorption on soil subsurface is one of the most important processes that allow the dye transport (Jedidi et al., 2020). The dye tracer Brilliant Blue FCF (C.I. Food Blue 2) has been applied (Forrer et al., 2000) to provide information on both the longitudinal and the lateral spreading of solutes in the soil, e.g., azo dyes in solutions that flow through the root zone.

In soils, azo compounds cause a decrease in microbial population and lead to a reduction in the enzyme activity (Krishnamoorthy et al., 2021); their presence acts to degrade the soil microbes. Azo dyes used in textile applications are key xenobiotic compounds bringing drastic changes in the microbial communities and adversely affecting soil ecosystems (Krishnamoorthy et al., 2021). Large concentrations of xenobiotics may be found in food chains and can persist for generations; in the case of synthetic dyes, the complete disappearance of color does not necessarily mean that the organic dyestuff molecular structures have been completely removed. Partial dye degradation may produce undesirable secondary pollutants that might be more toxic than their parent aromatic azo compound. By contrast, the cultivation of dye plants and the process of extraction and application of natural dyes pose no problems with the disposal of wastes from this industry; these may be applied in agricultural fields, i.e., the organic waste generated becomes an ideal biofertilizer.

When untreated textile sludge is applied to soils or crop lands, several of its ingredients pollute the soil clogging the pores followed by loss of fertility and productivity. Moreover, in a soil with high dye content, the decrease in the number of microorganisms causes a further reduction in soil productivity (Zheng et al., 2023), this also being a function of the potentially harmful effects on soil fertility arising from both the accumulation and degradation of azo dyes in soil systems; as the toxic effect of dyes ultimately leads to the death of soil microorganisms, plant growth is hampered. In short, textile dyes significantly reduce soil microbial activity, diversity, and richness of soil bacterial community (Zheng et al., 2023); these inhibition effects of an organic dyestuff may continue after the complete reduction of the parent dye molecule.

As already stated, azo dyes show inhibitory effects on plant growth promoting rhizobacteria (Imran et al., 2015); also, nitrogen-use-efficiency of plants is restricted by azo dye pollution with further reduction in productivity. Chemical pollution of agricultural land by organic dyes may lead to iron deficiency of crops grown in dye-contaninated soils (Zhou et al., 2004). Azo dyes may enter the food chain prior to their degradation; it has been observed (Imran et al., 2015) that large amounts of them are recoverable from soils after two weeks. There is direct evidence for dye uptake by plants and transport of accumulated dyes within plant tissues has been reported (Zhou, 2001). Finally, certain dyestuffs can cause irreparable damage to human health in addition to their toxicity to cultivation organisms.

The above discussion makes it possible to predict the fundamental factors determining both the dye migration and adsorption onto soils (Smaranda et al., 2017), as well as to design sustainable methods and practices for enhancing agricultural land conditions (Fragouli et al., 2023), promoting biodiversity and soil nutrition. Dye adsorption onto perlites seems to be one of these practices.

## Conclusions

Adsorption of the anionic dye C.I. Acid Black 1 from aqueous solutions on raw perlite and expanded perlite was investigated and the applicability of several adsorption isotherms was examined. Despite the like charges of perlite and dye, and the diversity of adsorption sites due to the non-uniformity of surface, dye anions insert, even though the dye-ion localization onto perlite decreases with temperature. The adsorption capacity of both perlites is increased with temperature; expanded perlite’s adsorption efficiency is slightly higher, a fact attributed to its open structure compared with the raw material (Roulia & Vassiliadis, 2008; Roulia et al., 2003). Adsorption of C.I. Acid Black 1 is controlled by the non-uniformity of perlites, because the surface heterogeneity can change the mechanism of dye condensation and may involve dyestuff interactions in the cooperative sense. Thus, dye-treated perlites satisfy both the Henderson and Halsey equations. The surface of perlite can be represented as a union of patches, characterized by their adsorption energy; the size of patches and the lateral interactions are important for describing the patchwise heterogeneity. Therefore, the applicability of BET model to perlites results from the patchwise surface topography that promotes the development of local dye supersaturations. As adsorption takes place only on a few highly active sites of perlites and dye “islands” are created, the isotherms of both perlites fit the Freundlich equation. A constant monolayer adsorption capacity value is observed for both adsorbents due to the absence of Coulombic interactions between the adsorbate and the substrate. Normally, adsorption on a uniform surface proceeds by the Langmuir equation and its fit in the AB 1–perlite system indicates the formation of a dye monolayer; however, only a minor surface portion of perlites is available for adsorption. In contrast to Langmuir model that assumes a single binding energy, Temkin suggests that the heat of adsorption in a layer decreases linearly with increasing surface coverage as a result of the adsorbate–adsorbate interactions and here the heterogeneity of binding is an important key that allows the best fit of experimental data to the Temkin isotherm for perlites.

C.I. Acid Black 1 is toxic, lethal to soil microorganisms, resulting in lower agricultural productivity. As AB 1 was found to be adsorbable on perlites, it can be subsequently removed from organic wastes and, thus, C.I. Acid Black 1 residues in agricultural soils can either be minimized or eliminated.

## References

[CR1] Abdullah H, Kuo D-H (2015). Photocatalytic performance of Ag and CuBiS_2_ nanoparticle-coated SiO_2_@TiO_2_ composite sphere under visible and ultraviolet light irradiation for azo dye degradation with the assistance of numerous nano p–n diodes. The Journal of Physical Chemistry C.

[CR3] Angelova R, Baldikova E, Pospiskova K, Safarikova M, Safarik I (2017). Magnetically modified sheaths of * Leptothrix * sp. as an adsorbent for amido black 10B removal. Journal of Magnetism and Magnetic Materials.

[CR4] Balachandran S, Swaminathan M (2012). Facile fabrication of heterostructured Bi_2_O_3_–ZnO photocatalyst and its enhanced photocatalytic activity. The Journal of Physical Chemistry C.

[CR5] Banisheykholeslami F, Hosseini M, Darzi GN (2021). Design of PAMAM grafted chitosan dendrimers biosorbent for removal of anionic dyes: Adsorption isotherms, kinetics and thermodynamics studies. International Journal of Biological Macromolecules.

[CR6] Bensalah J (2024). Removal of the textile dyes by a resin adsorbent polymeric: Insight into optimization, kinetics and isotherms adsorption phenomenally. Inorganic Chemistry Communications.

[CR7] Biyada S, Merzouki M, Dėmčėnko T, Vasiliauskienė D, Ivanec-Goranina R, Urbonavičius J, Marčiulaitienė E, Vasarevičius S, Benlemlih M (2021). Microbial community dynamics in the mesophilic and thermophilic phases of textile waste composting identified through next-generation sequencing. Scientific Reports.

[CR8] Brunauer S, Emmett PH, Teller E (1938). Adsorption of gases in multimolecular layers. Journal of the American Chemical Society.

[CR9] Cerofolini GF, Rudziński W, Rudziński W, Steele WA, Zgrablich G (1997). Theoretical principles of single- and mixed-gas adsorption equilibria on heterogeneous solid surfaces. Equilibria and dynamics of gas adsorption on heterogeneous solid surfaces.

[CR10] Forrer I, Papritz A, Kasteel R, Flühler H, Luca D (2000). Quantifying dye tracers in soil profiles by image processing. European Journal of Soil Science.

[CR11] Fragouli PG, Roulia M, Vassiliadis AA (2023). Macromolecular size and architecture of humic substances used in the dyes’ adsorptive removal from water and soil. Agronomy.

[CR12] Freundlich H (1907). Über die adsorption in Lösungen. Zeitschrift Für Physikalische Chemie.

[CR13] Galán J, Rodríguez A, Gómez JM, Allen SJ, Walker GM (2013). Reactive dye adsorption onto a novel mesoporous carbon. Chemical Engineering Journal.

[CR14] George G, Ealias AM, Saravanakumar MP (2024). Advancements in textile dye removal: A critical review of layered double hydroxides and clay minerals as efficient adsorbents. Environmental Science and Pollution Research.

[CR15] Georgiou D, Kalis M, Patermarakis G, Vassiliadis AA (2017). Destruction of azo-reactive dyes by ozonation and the synergetic effect of a radio-frequency alternating electric field inductance device. Current Trends in Fashion Technology and Textile Engineering.

[CR16] Ghanbari M, Salavati-Niasari M (2018). Tl_4_CdI_6_ nanostructures: Facile sonochemical synthesis and photocatalytic activity for removal of organic dyes. Inorganic Chemistry.

[CR17] Giles CH, MacEwan TH, Nakhwa SN, Smith D (1960). Studies in adsorption. Part XI. A system of classification of solution adsorption isotherms, and its use in diagnosis of adsorption mechanisms and in measurement of specific surface areas of solids. Journal of the Chemical Society.

[CR18] Gordon PF, Gregory P (1987). Organic chemistry in colour.

[CR19] Gregg SJ, Sing KSW (1982). Adsorption, surface area and porosity.

[CR20] Hadi M, Samarghandi MR, McKay G (2010). Equilibrium two-parameter isotherms of acid dyes sorption by activated carbons: Study of residual errors. Chemical Engineering Journal.

[CR21] Halsey G (1948). Physical adsorption on non-uniform surfaces. The Journal of Chemical Physics.

[CR22] Harkins WD, Jura G (1944). Surfaces of solids. XIII. A vapor adsorption method for the determination of the area of a solid without the assumption of a molecular area, and the areas occupied by nitrogen and other molecules on the surface of a solid. Journal of the American Chemical Society.

[CR23] He C, Hu X (2011). Anionic dye adsorption on chemically modified ordered mesoporous carbons. Industrial and Engineering Chemistry Research.

[CR24] Henderson SM (1952). A basic concept of equilibrium moisture. Agricultural Engineering.

[CR25] Imran M, Shaharoona B, Crowley DE, Khalid A, Hussain S, Arshad M (2015). The stability of textile azo dyes in soil and their impact on microbial phospholipid fatty acid profiles. Ecotoxicology and Environmental Safety.

[CR26] Jedidi A, Kraiem A, Dardouri S, Marcoux M, Sghaier J (2020). Adsorption of dye on a tunisian unsaturated layered soil: Physical and numerical modeling. Eurasian Soil Science.

[CR27] Jia YF, Xiao B, Thomas KM (2002). Adsorption of metal ions on nitrogen surface functional groups in activated carbons. Langmuir.

[CR28] Kandelbauer A, Cavaco-Paulo A, Gübitz GM, Christie RM (2007). Biotechnological treatment of textile dye effluent. Environmental Aspects of textile dyeing.

[CR29] Kazamias G, Roulia M, Kapsimali I, Chassapis K (2017). Innovative biocatalytic production of soil substrate from green waste compost as a sustainable peat substitute. Journal of Environmental Management.

[CR30] Khamis F, Hegab HM, Banat F, Arafat HA, Hasan SW (2024). Comprehensive review on ph and temperature-responsive polymeric adsorbents: Mechanisms, equilibrium, kinetics, and thermodynamics of adsorption processes for heavy metals and organic dyes. Chemosphere.

[CR31] Khoshraftar Z, Masoumi H, Ghaemi A (2023). On the performance of perlite as a mineral adsorbent for heavy metals ions and dye removal from industrial wastewater: A review of the state of the art. Case Studies in Chemical and Environmental Engineering.

[CR32] Kousha M, Daneshvar E, Sohrabi MS, Koutahzadeh N, Khataee AR (2012). Optimization of C.I. acid black 1 biosorption by * Cystoseira indica * and * Gracilaria persica * biomasses from aqueous solutions. International Biodeterioration and Biodegradation.

[CR33] Krishnamoorthy R, Choudhury AR, Jose PA, Suganya K, Senthilkumar M, Prabhakaran J, Gopal NO, Choi J, Kim K, Anandham R, Sa T (2021). Long-term exposure to azo dyes from textile wastewater causes the abundance of *Saccharibacteria* population. Applied Sciences.

[CR34] Kyzioł-Komosińska J, Rosik-Dulewska C, Dzieniszewska A, Pająk M (2011). Compost as biosorbent for removal of acid dyes from the wastewater generated by the textile industry. Archives of Environmental Protection.

[CR35] Langmuir I (1918). The adsorption of gases on plane surfaces of glass, mica and platinum. Journal of the American Chemical Society.

[CR36] Lima EC, Hosseini-Bandegharaei A, Moreno-Piraján JC, Anastopoulos I (2019). A critical review of the estimation of the thermodynamic parameters on adsorption equilibria. Wrong use of equilibrium constant in the Van't Hoff equation for calculation of thermodynamic parameters of adsorption. Journal of Molecular Liquids.

[CR37] Liu Q, Yang B, Zhang L, Huang R (2015). Adsorption of an anionic azo dye by cross-linked chitosan/bentonite composite. International Journal of Biological Macromolecules.

[CR38] Martin RW, Baillod CR, Mihelcic JR (2005). Low-temperature inhibition of the activated sludge process by an industrial discharge containing the azo dye acid black 1. Water Research.

[CR39] Mathew RT, Cooney RP, Zujovic Z, Doyle C, Wheelwright W, de Silva K (2018). A sustained release anchored biocide system utilizing the honeycomb cellular structure of expanded perlite. ACS Applied Bio Materials.

[CR40] Miranda ARL, Mendes LW, Lemos LN, Antunes JEL, Amorim MR, Melo VMM, de Melo WJ, Van den Brink PJ, Araujo ASF (2019). Dynamics of archaeal community in soil with application of composted tannery sludge. Scientific Reports.

[CR41] Murzin DY (2019). On the scientific heritage of Mikhail Isaakovich Temkin. Kinetics and Catalysis.

[CR42] Nethaji S, Sivasamy A (2011). Adsorptive removal of an acid dye by lignocellulosic waste biomass activated carbon: Equilibrium and kinetic studies. Chemosphere.

[CR43] Painer F, Baldermann A, Gallien F, Eichinger S, Steindl F, Dohrmann R, Dietzel M (2022). Synthesis of zeolites from fine-grained perlite and their application as sorbents. Materials.

[CR44] Pająk M, Dzieniszewska A, Kyzioł-Komosińska J (2019). Sorption of acid black 1 dye onto bentonite – equilibrium and kinetic studies. Journal of Environmental Science and Health A.

[CR45] Paprocki A, dos Santos HS, Hammerschitt ME, Pires M, Azevedo CMN (2010). Ozonation of azo dye acid black 1 under the suppression effect by chloride ion. Journal of the Brazilian Chemical Society.

[CR46] Patermarakis G, Vassiliadis AA (2023). Mechanism of C.I. reactive red 120 uptake from solution by anodic alumina films. Current Trends in Fashion Technology and Textile Engineering.

[CR47] Peng X, Hu X, Fu D, Lam FLY (2014). Adsorption removal of acid black 1 from aqueous solution using ordered mesoporous carbon. Applied Surface Science.

[CR48] Priya B, Uma L, Ahamed AK, Subramanian G, Prabaharan D (2011). Ability to use the diazo dye, C.I. acid black 1 as a nitrogen source by the marine cyanobacterium * Oscillatoria curviceps * BDU92191. Bioresource Technology.

[CR2] Qurrat-ul-Ain, Khatoon J, Shah MR, Malik MI, Khan IAT, Khurshid S, Naz R (2019). Convenient pH-responsive removal of acid black 1 by green L-histidine/iron oxide magnetic nanoadsorbent from water: Performance and mechanistic studies. RSC Advances.

[CR49] Roulia M, Vassiliadis AA (2005). Interactions between C.I. basic blue 41 and aluminosilicate sorbents. Journal of Colloid and Interface Science.

[CR50] Roulia M, Vassiliadis AA (2008). Sorption characterization of a cationic dye retained by clays and perlite. Microporous and Mesoporous Materials.

[CR51] Roulia M, Vassiliadis AA (2021). Water purification by potassium humate–C.I. basic blue 3 adsorption-based interactions. Agronomy.

[CR52] Roulia M, Chassapis K, Fotinopoulos C, Savvidis T, Katakis D (2003). Dispersion and sorption of oil spills by Emulsifier-modified expanded perlite. Spill Science and Technology Bulletin.

[CR53] Roulia M, Chassapis K, Kapoutsis JA, Kamitsos EI, Savvidis T (2006). Influence of thermal treatment on the water release and the glassy structure of perlite. Journal of Materials Science.

[CR54] Roulia M, Mavromoustakos T, Vassiliadis AA, Mali G (2014). Distinctive spectral and microscopic features for characterizing the three-dimensional local aluminosilicate structure of perlites. The Journal of Physical Chemistry C.

[CR55] Sellergren B, Shea KJ (1995). Origin of peak asymmetry and the effect of temperature on solute retention in enantiomer separations on imprinted chiral stationary phases. Journal of Chromatography A.

[CR56] Sepúlveda L, Fernández K, Contreras E, Palma C (2004). Adsorption of dyes using peat: Equilibrium and kinetic studies. Environmental Technology.

[CR57] Shaul GM, Holdsworth TJ, Dempsey CR, Dostal KA (1991). Fate of water soluble azo dyes in the activated sludge process. Chemosphere.

[CR58] Sivakumar V, Swaminathan G, Rao PG (2005). Studies on the influence of power ultrasound on dye penetration in leather dyeing using photomicrographic analysis. Journal of Microscopy.

[CR59] Smaranda C, Popescu M-C, Bulgariu D, Mǎluţan T, Gavrilescu M (2017). Adsorption of organic pollutants onto a romanian soil: Column dynamics and transport. Process Safety and Environmental Protection.

[CR60] Smith SE (1947). The sorption of water vapor by high polymers. Journal of the American Chemical Society.

[CR61] Smith TP, Zaklika KA, Thakur K, Walker GC, Tominaga K, Barbara PF (1991). Spectroscopic studies of excited-state intramolecular proton transfer in 1-(acylamino)anthraquinones. The Journal of Physical Chemistry.

[CR62] Stock M, Dunn S (2012). Influence of the ferroelectric nature of lithium niobate to drive photocatalytic dye decolorization under artificial solar light. The Journal of Physical Chemistry C.

[CR63] Sun D, Zhang X, Wu Y, Liu X (2010). Adsorption of anionic dyes from aqueous solution on fly ash. Journal of Hazardous Materials.

[CR64] Temkin MI (1979). The kinetics of some industrial heterogeneous catalytic reactions. Advances in Catalysis.

[CR65] Temkin MI, Pyzhev V (1940). Kinetics of ammonia synthesis on promoted iron catalysts. Acta Physicochimica U.r.s.s. (in Russian).

[CR66] Tovbin YK (2019). Development of the ideas of M. I. temkin in physical chemistry. Kinetics and Catalysis.

[CR67] Wang Y, Xia G, Wu C, Sun J, Song R, Huang W (2015). porous chitosan doped with graphene oxide as highly effective adsorbent for methyl orange and amido black 10B. Carbohydrate Polymers.

[CR68] Wuhrmann K, Mechsner K, Kappeler T (1980). Investigation on rate – determining factors in the microbial reduction of azo dyes. European Journal of Applied Microbiology and Biotechnology.

[CR69] Yariv S, Cross H (1979). Geochemistry of colloid systems.

[CR70] Zhao X, Bu X, Wu T, Zheng S-T, Wang L, Feng P (2013). Selective anion exchange with nanogated isoreticular positive metal-organic frameworks. Nature Communications.

[CR71] Zhao J, Qu X, Wang J, Yan B (2019). Photophysical tuning of viologen-based metal-organic framework hybrids via anion exchange and chemical sensing on persulfate (S_2_O_8_^2−^). Industrial and Engineering Chemistry Research.

[CR72] Zheng H, Yang X, Meng K, Li S, Yu H, Peng Q, Zhang Y, Zhang X, Xu X, Zhang Y, Xu Z, Li L, Ying Q, Elsheery NI (2023). Textile dyes alter the bacterial community structure in contaminated soil. Journal of Soil Science and Plant Nutrition.

[CR73] Zhou Q (2001). Chemical pollution and transport of organic dyes in water–soil–crop systems of the chinese coast. Bulletin of Environmental Contamination and Toxicology.

[CR74] Zhou Q, Xu J, Cheng Y (2004). Inhibitory effects of reactive X-3B red dye (RRD) on iron uptake by three crops. Plant and Soil.

